# Supplementary material for: Molecular transitions in early progenitors during human cord blood hematopoiesis
Source: Mol Syst Biol. 2018 Mar 15;14(3):e8041. doi: 10.15252/msb.20178041 (PMC5852373; doi:10.15252/msb.20178041)
Supplement: Supplementary file 1 — Expanded View Figures PDF [file MSB-14-e8041-s001.pdf]

Expanded View Figures

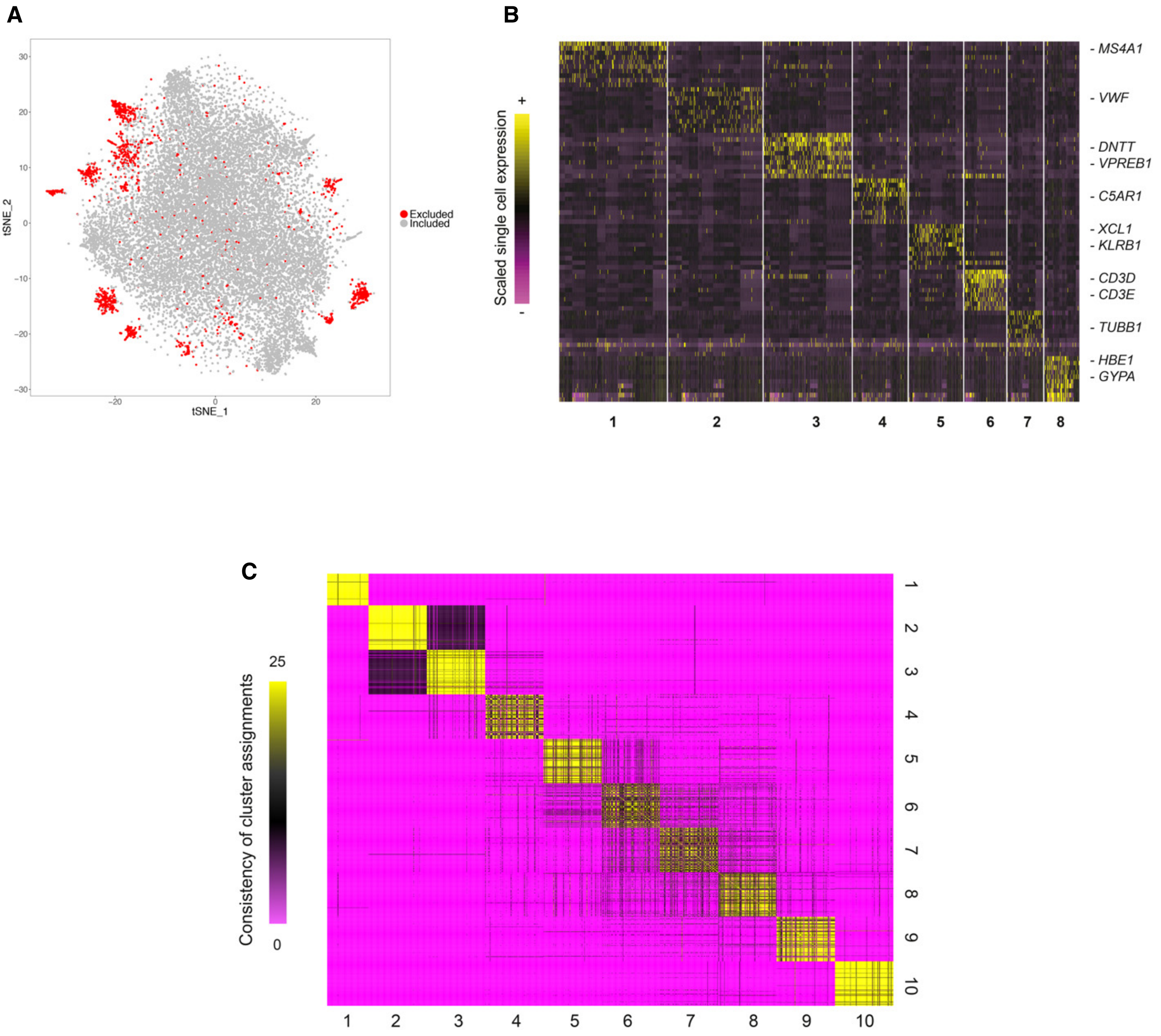

Figure EV1. Clustering of CD34^{+} single cells.

A A two-dimensional representation of 21,306 single cells from Drop-seq, using t-Distributed Stochastic Neighbor Embedding (tSNE). Excluded cells (shown in red) displayed markers of differentiated cells and were likely CD34$^{-/\text{low}}$ cells that passed through the CD34 column enrichment.

B Single-cell heatmap of cells excluded from downstream analysis and their signature markers. Expression values were scaled (*z*-scored) across single cells.

C Consensus matrix of results from 25 reclustering analyses using five resolution parameters (0.8–1.2) and five nearest-neighbor numbers *k* (15, 20, 25, 30, 35); entries indicate the number of analyses in which each pair of cells were assigned to the same cluster. Pairs of cells that clustered together in the full clustering also repeatedly cluster together across parameter values, with a median consistency of 0.81 (0.92 when considering clusters 2, 3, 9, and 10, which represent more differentiated cell states with increasingly clear boundaries).

© 2018 The Authors

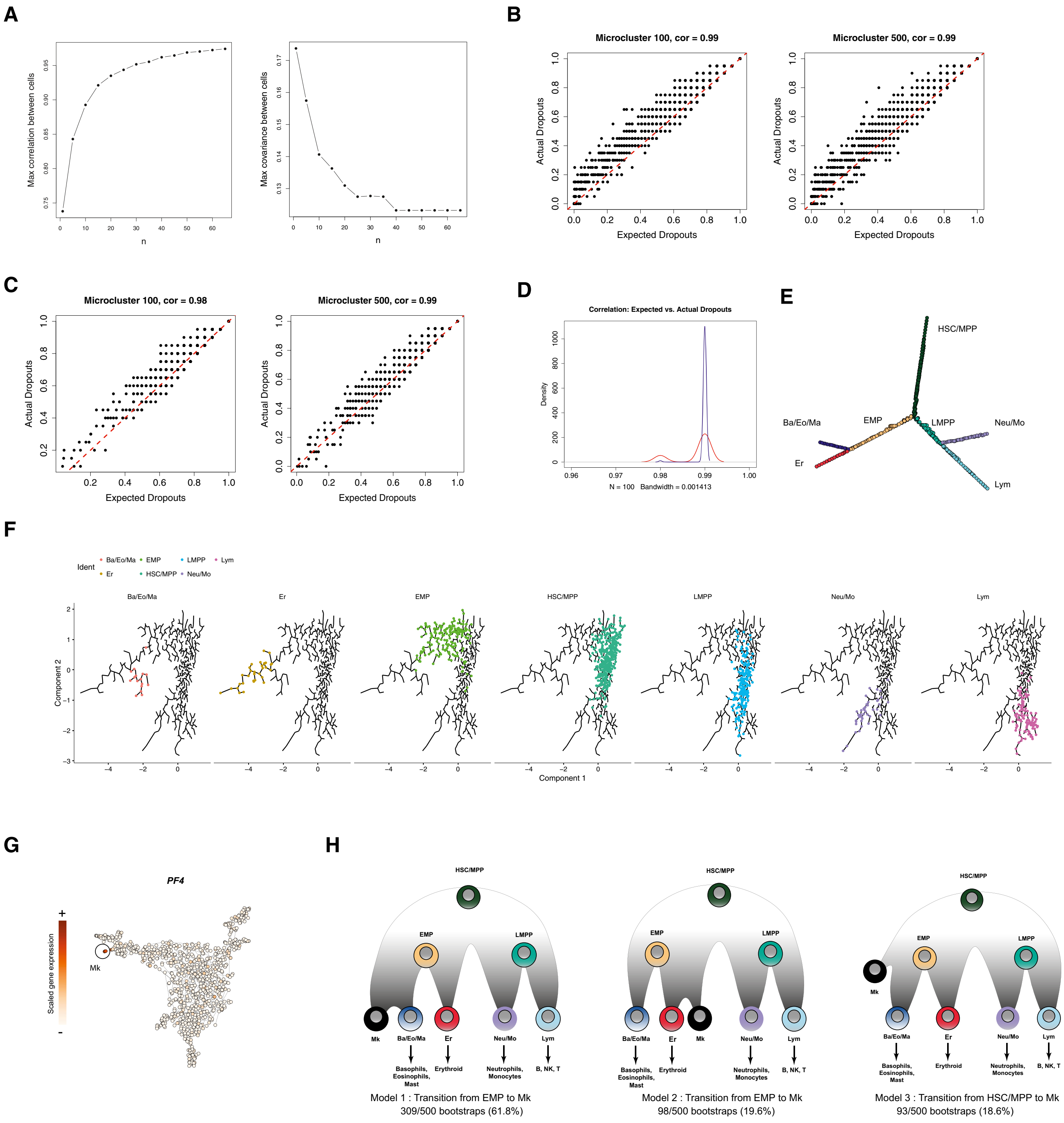

Figure EV2.

 © 2018 The Authors

◀ **Figure EV2. Reconstructing the hematopoietic hierarchy from micro-clusters.**

A The maximum pairwise correlation and covariance between micro-clusters after taking the average expression from n nearest single cells. We observe an increase in micro-cluster similarity with increasing values of n, approaching saturation at $n = 20$.

B, C Scatter plots showing the actual dropout rates per gene vs. the expected dropout rates predicted from stochastic Poisson distribution among single cells in one micro-cluster (title shows the Pearson correlation). Each point represents one gene, and examples are shown for two representative micro-clusters. Red lines in dash indicates $y = x$. (B) All detected genes are included in the analysis; (C) genes not used for clustering (non-variable genes) are shown.

D A density plot showing the distribution of correlations between expected dropouts and actual dropouts across single cells for 100 randomly selected micro-clusters. Blue: all genes; red: non-variable genes.

E A graph representation for the MST computed on 963 micro-clusters. This is the same MST as shown in Fig 2B, but here, the layout was computed using multidimensional scaling (MDS) based on the MST-derived distance matrix and adjusted using tkplot() in R, in order to more easily visualize the branching structure. Nodes are labeled in the same color scheme as in Fig 2B.

F Cellular hierarchy identified by running Monocle (Trapnell *et al*, 2014) on 960 micro-clusters (excluding Mk), specifying num_paths = 4 and reduction_method = “ICA”. The biological conclusions are fully consistent with our results.

G Scaled expression for PF4, a marker for Mk cells, across micro-clusters. Micro-clusters deriving from the Mk progenitor cluster C1 express PF4 and are circled and labeled.

H Results of 500 bootstraps of the MST construction process. All 500 bootstraps revealed identical hierarchical relationships for HSC and all four downstream lineages, with the exception of Mk progenitors. While most bootstraps revealed evidence for a transition from EMP to Mk cells, this was not uniform across data subsamples, demonstrating that the hierarchical placement of Mk progenitors was not entirely robust. We therefore focus on the remaining lineages, for which we identified consistent results across all bootstraps.

© 2018 The Authors

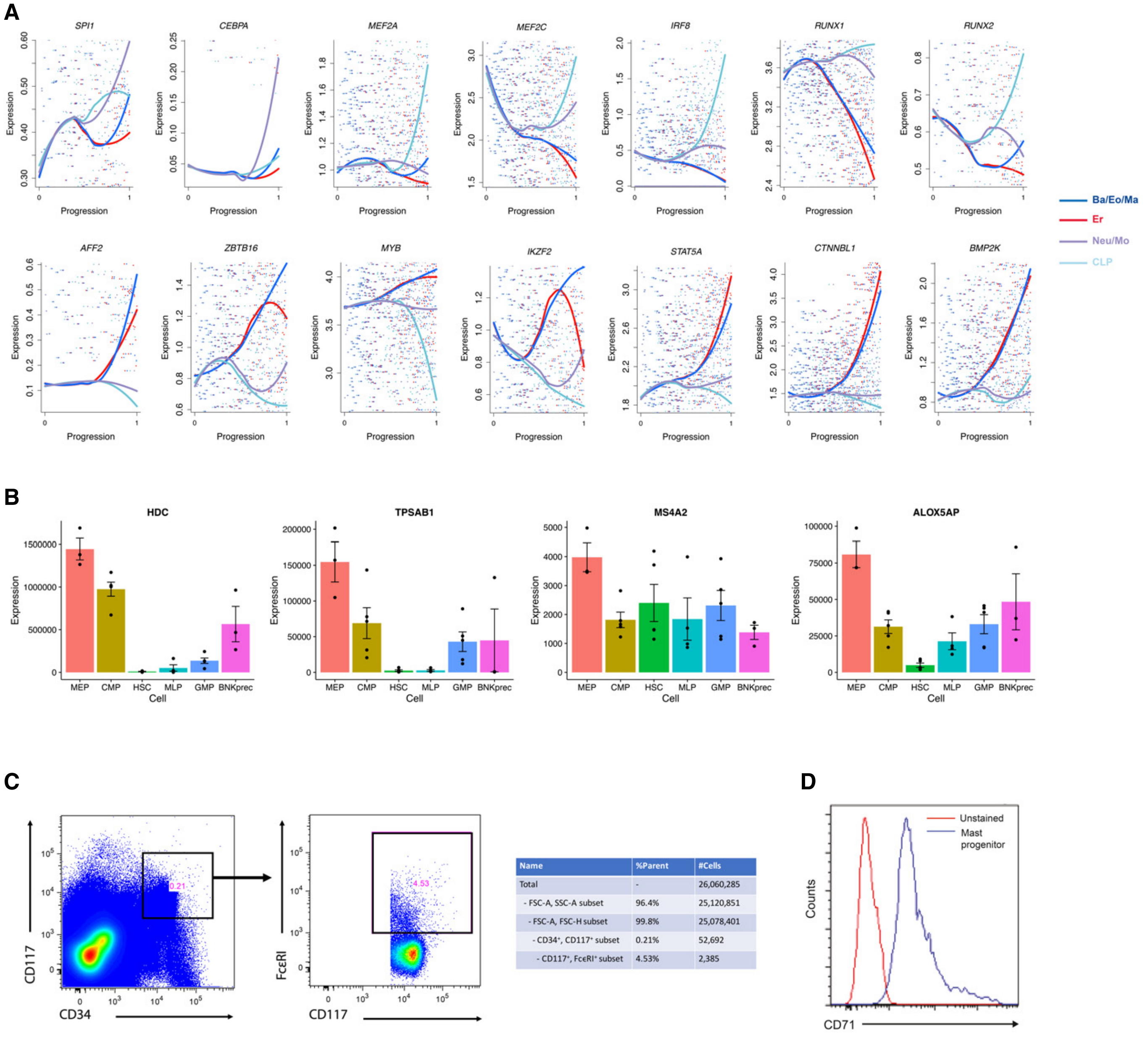

Figure EV3. Dynamic expression patterns for selected regulators and markers.

A Shown are the expression levels of canonical and novel regulators across the four trajectories, using the same color scheme as Fig 3B. *X*-axis reflects the developmental progression across each trajectory, representing the normalized distance from the MST root (Materials and Methods). A local polynomial regression with span = 0.9 was used for smoothing, with the underlying gene expression data shown as individual points.

B The normalized expression for four Ba/Eo/Ma markers, taken from a microarray dataset of reference progenitor populations (Laurenti *et al*, 2013). Error bars indicate standard errors across biological replicates (three replicates per population) from the original dataset. For all of these markers, expression is highest in the MEP population, indicating that our Ba/Eo/Ma population is traditionally mixed into the standard MEP gate.

C Gating strategy to enrich for mast cell progenitors ($CD34^{+}$ $CD117^{+}$ $Fc\varepsilon RI\alpha^{+}$) from human umbilical cord blood mononuclear cells, based on the sorting panel from Dahlin *et al* (2016). Accompanying table shows the proportion of each sorting gate and sorted cell numbers.

D Flow cytometry result showing CD71 protein expression on the surface of mast cell progenitors compared with unstained control, showing the enrichment of CD71 surface expression on mast cell progenitors.

 © 2018 The Authors

Figure EV4. Investigating early fate transitions in human bone marrow.

A Heatmap showing the conserved markers in annotated progenitor types from human bone marrow (Velten *et al*, 2017; left) or human umbilical cord blood (Drop-seq micro-clusters; right). The same heatmaps are in Fig 4C but here all gene names are shown.

B Enrichment scores for "primed" EMP genes, "primed" LMPP genes, "primed" lymphoid genes, and "primed" Neu/Mo genes (left to right) expression in cord blood $CD34^+$ micro-clusters, separated by progenitor types.

C Enrichment scores for "primed" EMP genes, "primed" LMPP genes, "primed" lymphoid genes, and "primed" Neu/Mo genes (left to right) expression in annotated bone marrow $CD34^+$ progenitors.

D Progenitor type proportions in bone marrow (black) and cord blood (gray).

E A consensus matrix showing the agreement between a random subset of 500 bone marrow cells from Velten *et al*, (2017), and the full bone marrow dataset when aligned to the cord blood micro-clusters. Results from the subsample are shown in rows, while results (for the subsampled cells) from the full analysis are shown in columns. A median "on-diagonal" consistency of 0.70 is achieved across all annotated cell states, where the consistency is higher among the four "endpoint" states (0.85).

F Results from PCA using accessible regions adjacent to genes from "primed" and "*de novo*" programs, showing the same structure as Fig 4E.

G "River" plots showing the quantitative changes in chromatin accessibility for "*de novo*" genes (using "primary peaks") during fate transitions to three downstream lineages from HSC.

H Heatmap showing *k*-means clustering for 324 peaks adjacent to "*de novo*" lymphoid genes (left), and 120 peaks for "*de novo*" EMP genes (right). For visualization, accessibilities are scaled (*z*-scored) across all experiments.

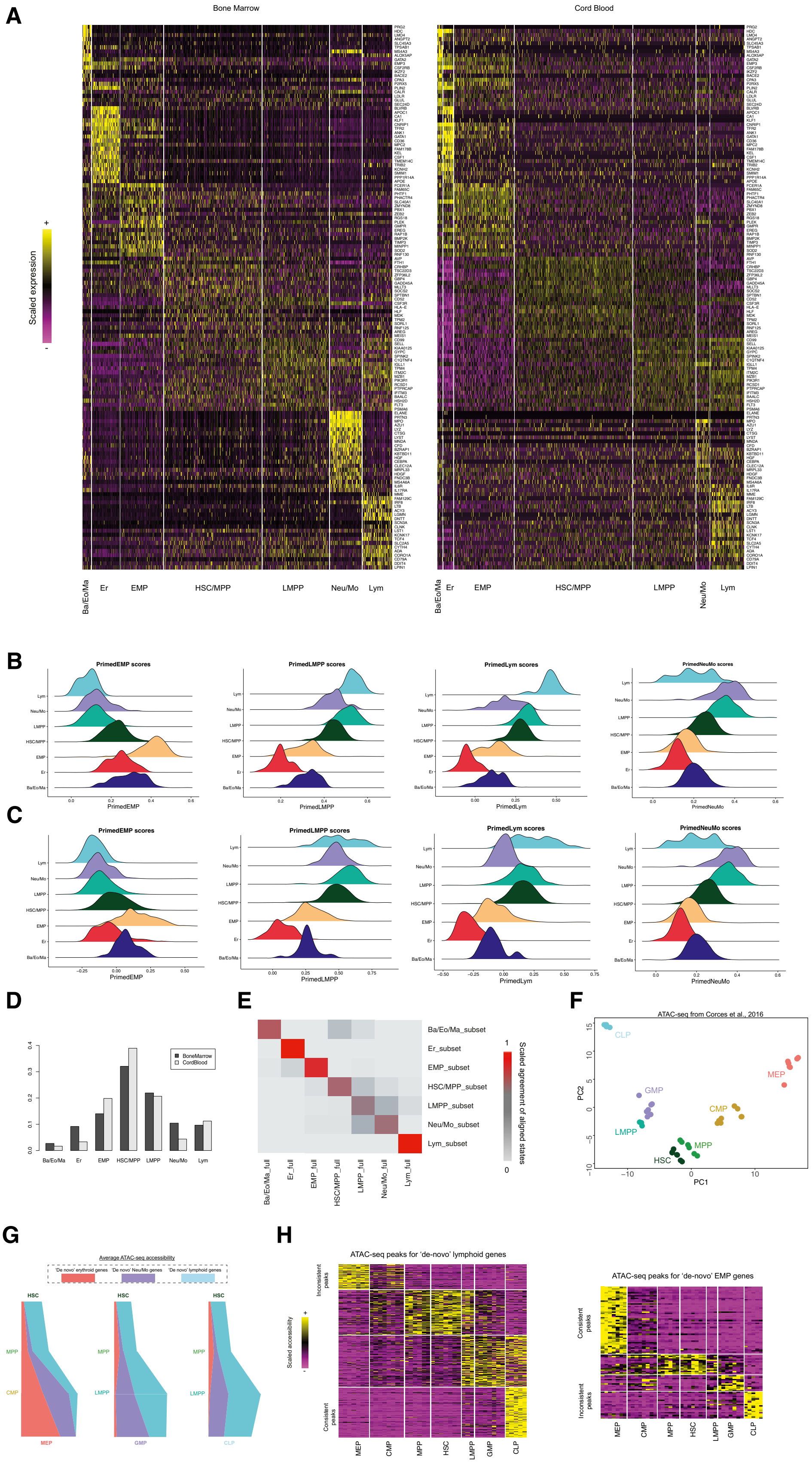

Figure EV4.

 © 2018 The Authors

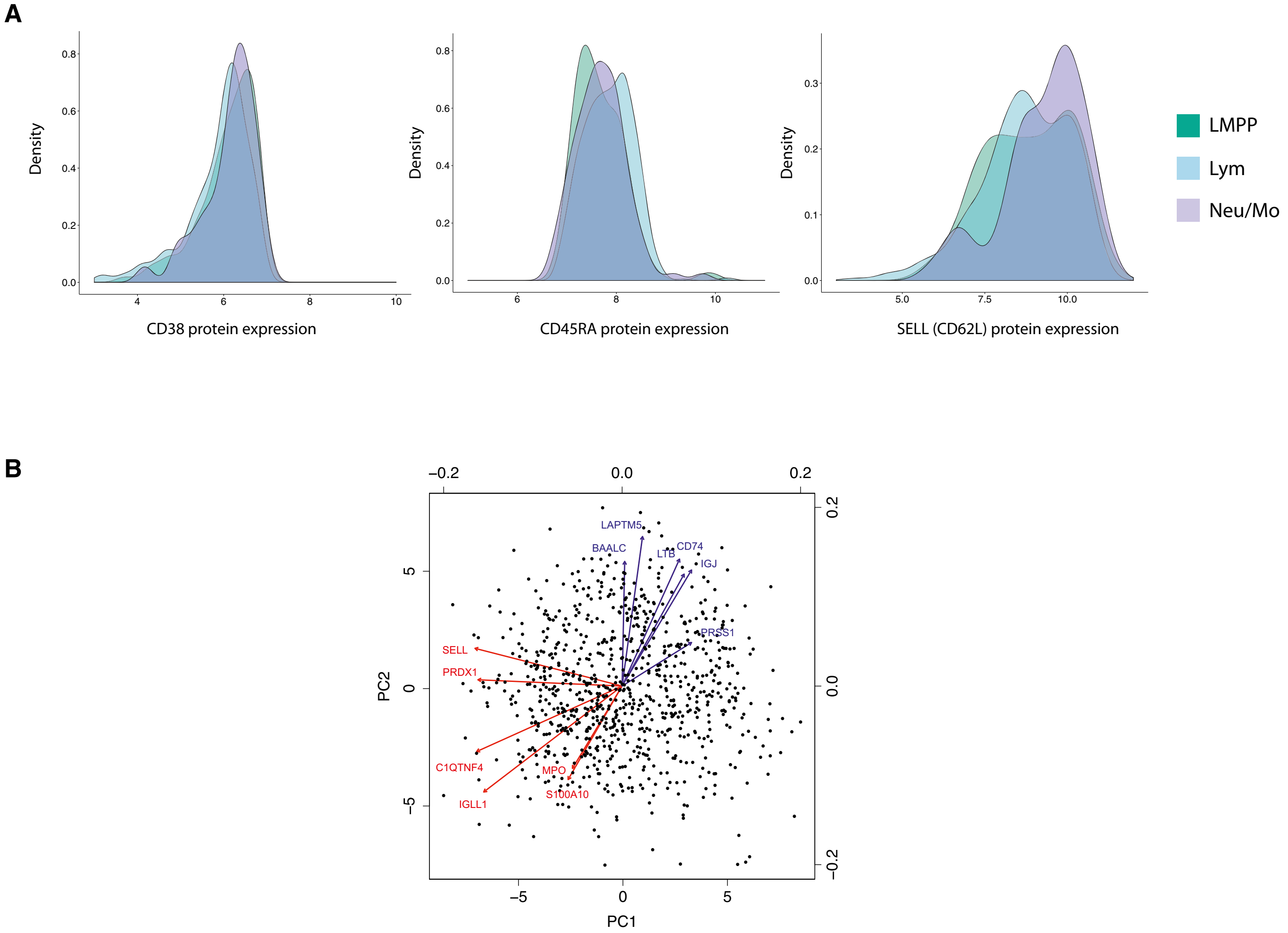

Figure EV5. Coupling immunophenotypes to Drop-seq data.

A Distribution of indexed protein levels for CD38, CD45RA and SELL (CD62L) in sorted CD34$^+$ CD38$^-$ CD45RA$^+$ cells. Protein expression is shown in log10 scale.

B A biplot showing results from principal component analysis (PCA) on 865 sorted CD34$^+$ CD38$^-$ CD45RA$^+$ cells, using genes from the dynamic expression programs identified in Fig 3A. Points represent single cells plotted on PC embeddings. The arrows show PC loadings for the top 12 genes in PC1 and PC2. Genes in blue are members of lymphoid gene modules, while red genes are members of myeloid gene modules.

© 2018 The Authors

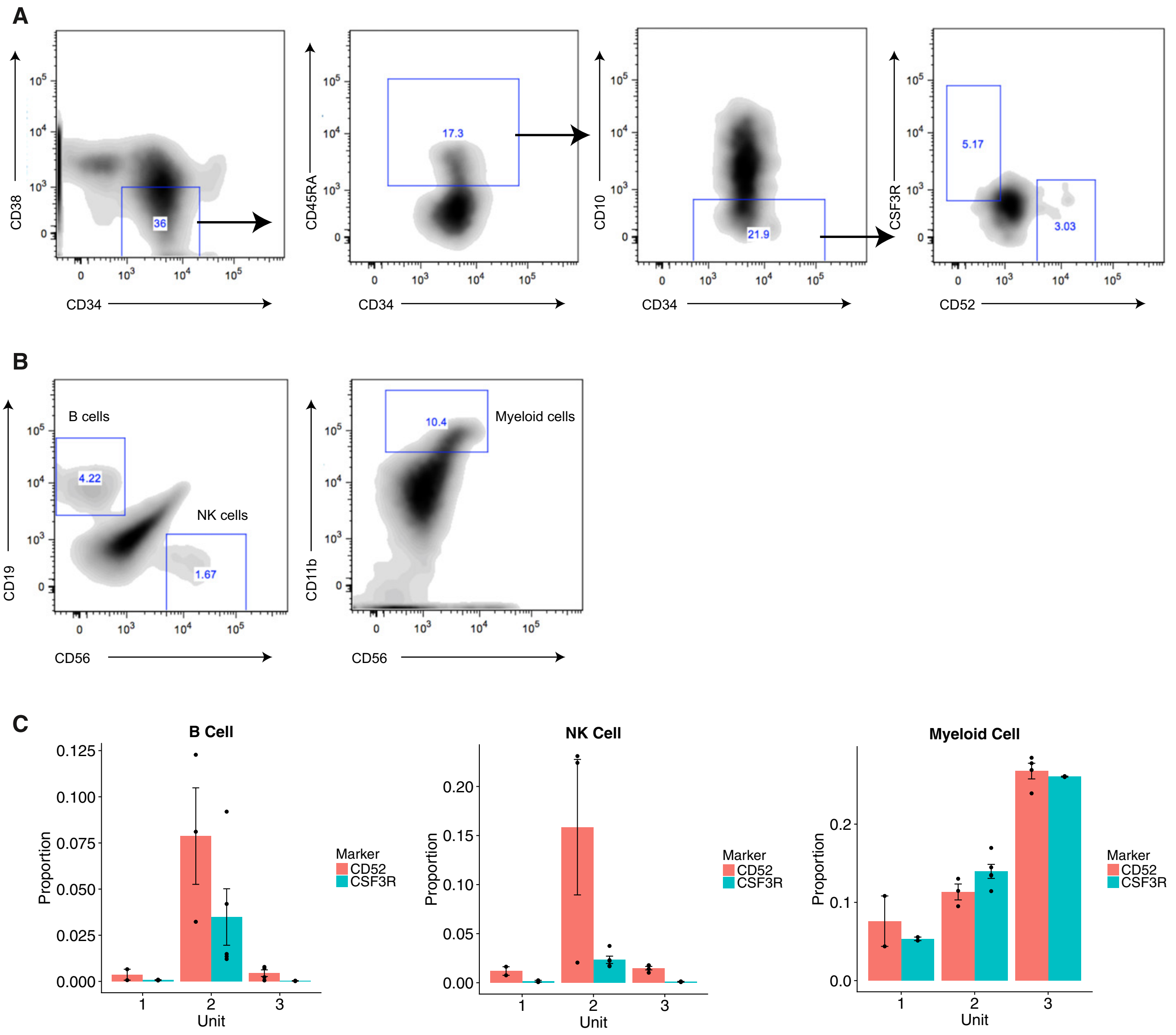

Figure EV6. ***In vitro* differentiation using MS5-MBN assay (Laurenti *et al*, 2013).**

A Gating strategy of sorting LMPP subsets for MS5-MBN differentiation assay, by further gating on CD52 and CSF3R for the canonical LMPP sorting gate ($CD34^+$ $CD38^-$ $CD45RA^+$ $CD10^-$).

B Gating strategy for identifying $CD19^+$ $CD56^-$ B cells, $CD19^-$ $CD56^+$ NK cells, $CD56^-$ $CD11b^+$ myeloid cells 3 weeks after initial seeding of 250–300 LMPP subsets.

C Bar plots showing the proportion of differentiated B, NK and myeloid cells among all $CD45^+$ human cells in all three cord blood units. Data are shown in mean ± standard error of cell type proportions across 2–5 replicates per cord blood unit.

© 2018 The Authors
